# Evidence for use of a healthy relationships assessment tool in the CHARISMA pilot study

**DOI:** 10.1371/journal.pone.0261526

**Published:** 2021-12-31

**Authors:** Elizabeth E. Tolley, Andres Martinez, Seth Zissette, Thesla Palanee-Phillips, Florence Mathebula, Siyanda Tenza, Miriam Hartmann, Elizabeth T. Montgomery

**Affiliations:** 1 FHI 360, Behavioral, Epidemiological and Sciences, Durham, NC, United States of America; 2 Maternal and Child Health Dept, University of North Carolina, Chapel Hill, NC, United States of America; 3 Wits Reproductive Health and HIV Institute, Faculty of Health Sciences, University of the Witwatersrand, Johannesburg, South Africa; 4 RTI, Women’s Global Health Imperative, Berkeley, CA, United States of America; University of North Carolina at Chapel Hill, UNITED STATES

## Abstract

**Introduction:**

The CHARISMA intervention, nested within the MTN-025/HOPE vaginal ring trial in Johannesburg, South Africa, seeks to facilitate women’s use of HIV prevention products by promoting partner dialogue and mitigating intimate partner violence (IPV). We developed “HEART”, a lay counselor-administered relationship assessment tool, for the CHARISMA pilot. The five-scale tool assesses participants’ endorsement of Traditional Values (TV), her HIV Prevention Readiness (HPR) and levels of partner support (PS), abuse and control (PAC), and resistance to HIV prevention (PR), guiding decisions about which of three counselling modules to offer (partner communication/A; ring disclosure/B; and IPV prevention/C).

**Methods:**

We correlated baseline scores on HEART subscales with a) independent measures of relationship stability, disclosure and IPV to assess construct validity, and b) with specific modules offered to determine how HEART was used in the pilot. We examined changes in HEART scores at three and six months. Finally, we ran separate growth models for each subscale to examine changes in scores, accounting for partnership changes and counseling module(s) received.

**Results:**

Baseline HEART scores correlated as predicted among subscales and with other measures. Reliabilities for four subscales were 0.75 or higher. Women who disclosed study participation and ring use scored higher on PS and lower on PR. Women experiencing IPV scored lower on PS, and higher on PAC and PR. During the pilot, 82% of women received one and 17% received two or more modules; over half received the IPV module. Women with higher PAC and PR scores were more likely to receive the IPV than the communication or disclosure modules. Over time, the TV, PAC and PR scores decreased, and PS score increased. Receiving the IPV module was associated with a decreased PAC score.

**Conclusions:**

These data offer preliminary evidence for HEART construct and predictive validity and support its further evaluation to guide implementation and monitor the impact of the CHARISMA intervention in a randomized controlled evaluation.

## Introduction

Women worldwide continue to be at risk of HIV, with younger southern African women often at highest risk [1]. Women’s sources of risk are multiple, but often intertwined with the dynamics of their social and sexual relationships. In one cohort study of more than 1000 women in Cape Town, SA, women who experienced intimate partner violence (IPV) were 1.5 times more likely to acquire HIV, and women with lower levels of relationship power had higher rates of HIV infection than those with higher power [2]. In several trials evaluating new vaginal and oral pre-exposure prophylaxis (PrEP) HIV prevention products within these same populations, adherence has been low [3–6]. Low adherence in clinical trial settings has been attributed to concerns about using experimental products [7, 8]; alternative (non-product) motivations for trial participation, low perceived risk of HIV [9], and social and sexual challenges related to trial and product use [10, 11]. Such challenges reportedly include overcoming male partners’ distrust of trial objectives, their concerns about potential product-related side effects or about how product use and trial participation might disrupt existing relationship dynamics, including by facilitating a female partner’s promiscuity or encouraging greater female autonomy [12].

In demonstration studies in which product efficacy is known, adolescent girls and young women (AGYW) have shown interest in using oral PrEP. However, in several studies initial uptake remained low, and among those who initiated PrEP, adherence to and persistence of PrEP decreased over time [13–17]. For example, among more than 1200 HIV-uninfected Kenyan women who were screened for PrEP in the context of family planning services, 278 women initiated PrEP and fewer than half (114, or 41%) returned for at least one refill. Continuation at six months was 15% [16]. In qualitative interviews with Kenyan AGYW accessing PrEP through maternal child health and family planning clinics, negative reactions from male partners were frequently raised as a reason for non-use or discontinuation of PrEP [18]. In an oral PrEP demonstration study with serodiscordant couples, women reporting recent IPV were more likely to have low adherence [19, 20]. These studies suggest that the need to negotiate PrEP use and the potential for a partner’s lack of support, his resistance or active abuse remain barriers to women’s use of new prevention methods, even when discreet use is theoretically possible. To date, few interventions have been developed or evaluated that directly support women’s ability to engage (or not engage) their partners in the context of clinical trial participation or routine use of PrEP.

Addressing this gap, the Community Health clinic model for Agency in Relationships and Safer Microbicide Adherence (CHARISMA) intervention was developed to increase women’s agency to consistently and safely use ARV-based HIV prevention products such as vaginal microbicides and oral pre-exposure prophylaxis (PrEP), while also reducing their risk of intimate partner violence (IPV) and promoting healthy relationships [21]. CHARISMA was pilot tested at the Wits Reproductive Health and HIV Institute (Wits RHI) site in Johannesburg, South Africa. Women who had previously participated in the Phase 3 MTN-020/ ASPIRE trial (ClinicalTrials.gov number NCT01617096 and NCT02858037), evaluating the safety, acceptability and effectiveness of a monthly vaginal ring (VR) containing 25 mg of dapivirine and had then transitioned to the MTN-025/HIV Open-label Prevention Extension (HOPE) trial, which assessed the extended safety of and adherence to VR use were eligible to enroll. Women who agreed to participate in the six-month CHARISMA pilot intervention study received differentiated in-person counseling based on a baseline assessment of their primary relationship using the HEAlthy Relationship Assessment Tool–referred to as HEART; both HEART and the differentiated counseling module were delivered by a trained lay counselor. Counseling modules included: partner communication (A), ring disclosure (B) and IPV prevention (C).

The initial development and validation of HEART, which was conducted prior to CHARISMA pilot study implementation, has been described elsewhere [21, 22]. The purpose of this manuscript is to describe the performance and prospective validity of HEART, from which we can infer the tool’s utility to screen, monitor and/or intervene on women’s relationships within the context of PrEP delivery. In this manuscript, we use initial scale development data collected during formative work and data from the pilot intervention to examine how the pilot HEART subscales: 1) perform vis-à-vis the initial scale development survey, 2) correlate with other baseline measures describing pilot participants’ relationships, 3) were used to guide the counseling component of the intervention, and 4) performed over time. The results of these analyses informed the use of the HEART tool in a final randomized controlled effectiveness evaluation of the CHARISMA intervention (manuscripts forthcoming.)

## Methods

### Parent studies

Our analyses draw on two separate studies. In our *formative study*, conducted between April to September 2016 at Wits RHI, we administered a cross-sectional survey, consisting of 127 potential HEART items and relevant socio-demographic and risk behavior variables, to 309 women. Eligible women were aged 18 to 40, and either had prior experience as former trial participants (FTPs) in HIV prevention research or were trial-naïve participants (TNPs). While more fully described in a separate manuscript [22], the goal of the formative study was to identify constructs (e.g., partner support, abuse or other dimensions of partner relationships) and items (e.g., the specific questions or statements) that could characterize women’s primary relationships and be formalized in a counselor-administered tool to guide various modules offered as part of the CHARISMA intervention. As described in an earlier paper [21], potential items for the tool were drawn from existing index or scale measures of IPV [23–25], sexual power–including the Sexual Relationship Power Scale [26, 27], or partner support and decision-making [28]. Because many of the original measures comprised 10–40 items each, we conducted an exploratory scale development process to determine whether an abbreviated set of items, feasible for use in a clinic setting, might emerge that assessed the spectrum of partner dynamics, from supportive to abusive.

The *pilot CHARISMA intervention* was conducted from December 2016 to October 2018. Also described elsewhere [29], this intervention co-enrolled all 95 women at the Wits RHI site who were current participants in the HOPE open-label extension of the dapivirine vaginal ring. (An additional five participants enrolled in HOPE after the CHARISMA enrollment period had ended, so did not participate in the pilot). All participants in the pilot CHARISMA intervention were administered the HEART at baseline and then received a brief module on healthy relationships, which included counseling on forms of abuse and control. Depending on their HEART scores and information about whether they had disclosed study participation and/or product use to a partner, they also might have received a module on a) partner communication; b) ring disclosure; or c) IPV prevention. All women were also offered referrals to external services. A brief booster counseling session was provided to participants at month 1, or at three- and six-month visits if new partners or IPV were reported during follow-up. However, the HEART was administered at month 1 only to those participants who reported having a new partner. At months three and six the HEART was administered to all participants to measure any changes in attitudes or relationship behaviors, but not to guide delivery of additional counseling.

The participants enrolled in both the formative and pilot studies were recruited through the Wits RHI site in Johannesburg, South Africa. Both studies were reviewed and approved by the University of Witwatersrand Human research ethics committee (HREC). In addition, the formative study was reviewed and approved by FHI 360’s Protection of Human Subjects Committee. The RTI IRB deferred approval decisions to the HREC IRB for the pilot CHARISMA intervention. Participants in both studies provided written informed consent prior to enrollment.

### Measures

The HEART comprises five subscales and a total of 42 statements. All items are scored on a 6-point scale from strongly disagree (coded 1) to strongly agree (coded 6). The subscales were:

1. Traditional Values (TV): 13 items describing norms valuing masculinity (e.g., *A man should have the final say in all family matters; I think there is nothing a woman can do if her husband wants to have girlfriends)*;
2. Partner Support (PS): 10 items describing ways that the relationship with her partner is/is not supportive or harmonious (e.g., *My partner is as committed as I am to our relationship/ My partner does what he wants*, *even if I do not want him to)*;
3. Partner Abuse & Control (PAC): 9 items describing a partner’s psychologically or physically abusive behaviors or their outcomes (e.g., *My partner makes fun of me or humiliates me; My partner slaps*, *hits*, *kicks*, *or pushes me; I can’t seem to make good decisions about my life)*;
4. Partner Resistance to HIV Prevention (PR): 5 items describing partner’s unwillingness to talk about or use HIV prevention (e.g., *If I asked my partner to use a condom*, *he would get angry)*; and
5. HIV Prevention Readiness (HPR): 5 items expressing individual or joint readiness to use HIV prevention products (e.g., *Using an HIV prevention product shows that my partner and I care about each other)*.

In both studies, the HEART items were electronically administered by a researcher or counselor on a tablet. In the CHARISMA pilot, item responses within each subscale were summed to obtain a subscale score after replacing any missing item response by the mean of the available responses. The participants’ subscale scores were compared to the mean scores from the original formative study. Scores in the pilot CHARISMA intervention that were one or more standard deviations below the formative study mean (the benchmark) were determined to be “low” in terms of the construct, while those that were one or more standard deviations above the mean were determined to be “high”. All other scores fell into a “medium” category. The HEART tool produced a report noting which, if any, subscales were in the “risk zone”. For example, a PS summary score that was one or more standard deviations below the benchmark, or a PAC or PR score that was one or more standard deviations above the benchmark from the formative study, was considered a risk and flagged by the report, recommending that the counselor offer the IPV module to the participant. (The IPV module could also be offered at the discretion of the counselor, regardless of HEART score on these subscales. Counselors typically offered the IPV module for women in the moderate and high-risk categories.)

#### Other variables: Relationship stability

At baseline, participants were asked whether they had a primary partner in the last three months and, if so, whether this was the same partner they had during their previous trial participation, as well as whether they were living with their primary partner. *Disclosure*. At each visit, participants were asked whether their primary sex partner knew about their use a vaginal ring as part of this study. Response options were “Yes”, “No”, and “Not sure”. *IPV*. At each visit, women were asked a four-part question about whether their primary sex partner or any other current or previous partner had ever committed acts of physical or sexual violence against them within the past 3 months and was classified as experiencing IPV if a response of “yes” was given to any part of the question.

### Analytic approach

In this analysis, we used the CHARISMA pilot intervention data to prospectively validate the HEART. We compared the subscale mean scores, reliabilities and correlations to those from the original formative survey to assess whether the tool performed similarly in different samples (RQ1). We assessed construct validity by correlating the pilot study baseline scores on the HEART subscales with a) independent measures of relationship stability, disclosure and IPV to assess construct validity (RQ2), and b) specific modules offered to determine how HEART was used in the pilot (RQ3). We also fit simple and multivariable logistic regression models to further assess the strength of the associations between the HEART subscales and reports of IPV and disclosure of ring and study to partner. Finally, to examine the predictive validity of the tool, we examined changes in HEART scores at three and six months (follow-ups 1 and 2) and ran separate growth models for each subscale to describe changes in scores, accounting for changes in partner relationship and receipt of counseling modules (RQ4).

## Results

Women in the formative study included both trial-naïve participants (TNP) and those with former trial experience (FTP). As presented in Table 1, trial-naïve participants tended to be younger, less likely to have children or to earn their own income than those who had formerly or were currently participating in a clinical trial. Women participating in the CHARISMA pilot study were older than participants in the earlier formative study, but more similar in age, education status, having children and earning an income, to FTPs than to TNPs from the formative study.

**Table 1:** Sociodemographic characteristics of formative and pilot study samples.

	Formative Survey			Pilot
	ALL (n = 309)	TNP(n = 245)	FTP(n = 64)	FTP(n = 96)
Mean age in years, (range)	27 (18–46)	26 (18–46)	29 (19–46)	31 (21–48)
	*%*	*%*	*%*	*%*
Proportion of participants aged ≤ 25	52	57	34	26
Ever participated in HIV prevention clinical trial research	21	n/a	100	100
Type of trial:	n/a	n/a		
Vaginal ring			88	100
Vaginal gel			5	
Other (not oral PrEP)			8	
Have children	68	66	77	82
Highest level of education:				
Incomplete secondary, or less	28	28	24	30
Secondary, complete	37	34	48	46
Attended college or university	36	39	28	24
Earns an income	36	32	52	44

The results of our HEART validations are presented below, organized by four core research questions, that examine how the pilot HEART subscales: 1) perform vis-à-vis the initial scale development survey, 2) correlate with other baseline measures describing pilot participants’ relationships, 3) were used to guide the CHARISMA intervention, and 4) performed over time in the CHARISMA pilot intervention.

### Research question 1: How do pilot HEART subscales perform vis-à-vis the formative scale development survey?

**Fig 1:**
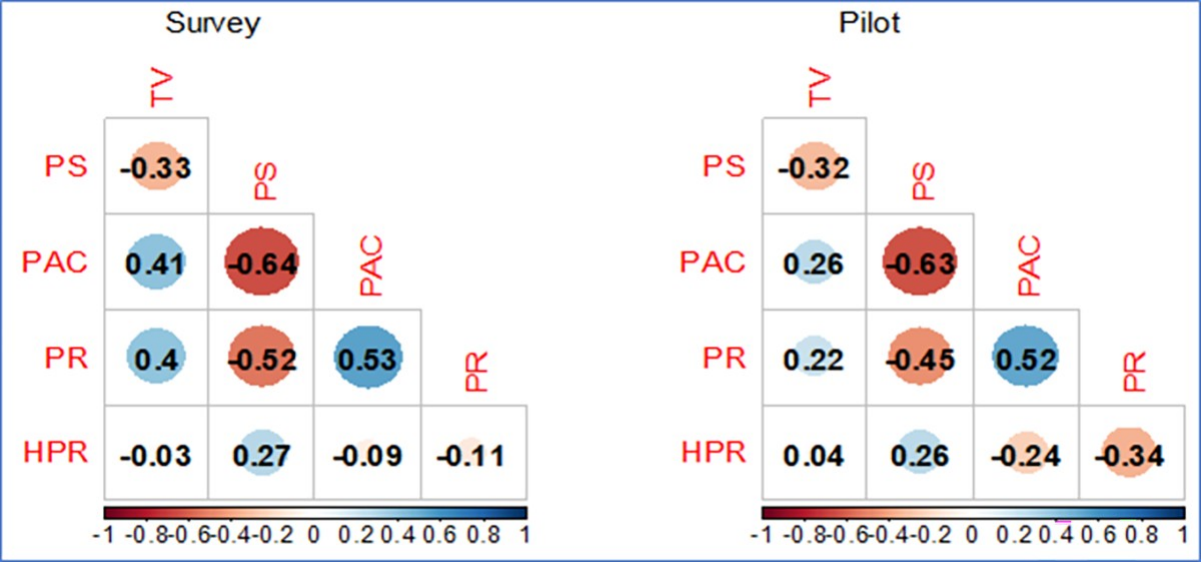
Comparison of subscale correlations for two samples. Caption: TV = Traditional Values; PS = Partner Support; PAC = Partner Abuse & Control; PR = Partner Resistance to HIV Prevention; HPR = HIV Prevention Readiness. Blue shading = positive correlation; Red shading = negative correlation. Larger circles and deeper shades = stronger correlations.

Fig 1 presents a side-by-side visual representation of subscale correlations between the formative and pilot study samples, with positive correlations represented in blue and negative correlations in red, and with larger circles and deeper shades of color representing stronger associations. Our analyses suggest the HEART subscales functioned similarly in the two samples.

First, correlations between the different subscales in the pilot study were similar in direction and strength to what we had predicted and found in the original formative survey. As shown in Fig 1, the PAC scale was strong and inversely correlated with PS (-0.64 in the formative survey and -0.63 in the pilot study) and strongly positively correlated with PR scores (0.53 and 0.52 respectively). The TV subscale showed a similar direction and level of correlation with PS in both studies, but the strength of positive correlations was stronger in the formative survey than in the pilot. In contrast, inverse relationships between HPR and several subscales (PAC and PR) were stronger in the pilot study.

Additionally, the subscale reliabilities in our pilot study were similar to those in the original survey (Table 2). The Cronbach Alpha coefficient, which measures how closely related individual items are to the subscale construct, was equal to or greater than 0.70 in the same four HEART subscales for both samples, demonstrating generally acceptable levels of internal reliability [30]. One exception, however, was the HPR reliability measure, which was borderline in the formative survey and even lower in the pilot study. (S1 table provides additional measures of reliability for two samples.)

**Table 2:** Comparison of mean scores and internal reliabilities for two samples.

Factor	# Items (range)	Mean (Std) Survey	Mean (Std) Pilot	α Survey	α Pilot
Traditional Values (TV)	13 (13–78)	31.7 (13.9)	26.2 (12.5)	0.84	0.84
Partner Support (PS)	10 (10–60)	46.2 (10.3)	49.0 (9.4)	0.81	0.79
Partner Abuse & Control (PAC)	9 (9–54)	19.5 (9.5)	15.3 (7.3)	0.81	0.74
Partner Resistance (PR)	5 (5–30)	10.5 (6.5)	8.5 (5.5)	0.80	0.83
HIV Prevention Readiness (HPR)	5 (5–30)	27.3 (3.7)	28.7 (2.4)	0.68	0.54

### Research question 2: How do HEART subscales correlate with other baseline measures describing pilot participants’ relationships?

In our formative study analysis [22], we predicted and confirmed that PAC and PR scales would be positively correlated with measures of IPV and negatively associated with disclosure. Similarly, we found that PS was negatively correlated with IPV measures, but positively correlated with disclosure. However, HEART associations with sociodemographic and relationship variables were less likely to match our predictions.

Among the pilot population, we found similar patterns of association. As shown in Table 3, women who reported any IPV had lower PS scores (e.g. 43.9 vs 49.6) and higher PAC and PR scores than women who did not report any IPV (e.g., 21.9 vs 14.4; 13.2 vs.7.9 respectively), with differences all large enough to be statistically significant (at the value *p < 0.1; ** p < 0.05; ***p < 0.01). Although women who reported any IPV also had higher TV scores, the difference was not statistically significant in this sample. With regards to disclosure, women who fully disclosed ring use and study participation to their partners had higher PS and lower PR scores, with marginally significant differences in mean scores (at p<0.1).

**Table 3:** Comparison of HEART mean scores by independent partner measures.

	Any IPV			Full Disclosure		
	No N = 83	Yes N = 12	Diff. (SE)	No N = 36	Yes N = 59	Diff. (SE)
Traditional Values (TV) (range 13–78)	25.6	29.7	‒4.02 (3.46)	26.1	26.2	‒0.08 (2.55)
Partner Support (PS) (range 10–60)	49.6	43.9	5.72* (3.03)	46.4	50.5	‒4.11* (2.11)
Partner Abuse & Control (PAC) (range 9–54)	14.4	21.9	‒7.54*** (2.77)	15.9	14.9	0.96 (1.56)
Partner Resistance (PR) (range 5–30)	7.9	13.2	‒5.39** (2.56)	10.2	7.5	2.76* (1.24)
HIV Prevention Readiness (HPR) (range 5–30)	28.6	28.7	‒0.05 (0.62)	28.2	28.8	‒0.60 (0.52)
	Aged ≤ 25 vs > 25			Living w/Partner		
	No N = 70	Yes N = 25	Diff. (SE)	No N = 58	Yes N = 37	Diff. (SE)
Traditional Values (range 13–78)	27.1	23.7	3.42 (2.9)	25.28	27.5	‒2.26 (2.68)
Partner Support (range 10–60)	49.6	46.9	2.76 (2.21)	49.1	48.6	0.48 (1.88)
Partner Abuse & Control (range 9–54)	14.5	17.8	‒3.3* (1.68)	14.2	17.1	‒2.92** (1.62)
Partner Resistance (range 5–30)	8.3	9.1	‒0.74 (1.28)	7.9	9.7	‒1.91* (1.14)
HIV Prevention Readiness (range 5–30)	28.6	28.6	0.08 (0.59)	28.7	28.5	0.13 (0.57)

* Associated probability value (p) < 0.1

** p < 0.05

***p < 0.01 from Student t-tests assuming unequal variances. One-sided hypothesis tests for all subscales except Traditional Values.

Younger women aged 18 to 25 scored higher on the PAC subscale than older women (p = .05). Women who reported living with a partner scored significantly higher in PAC and PR. All the differences in PS, PAC, PR and HPR are in the originally hypothesized direction, though the differences in some cases were not as large as expected.

To further explore the nature and strength of the relationships between the HEART scores and the three partner-related indicators (i.e., experiencing any IPV, full disclosure, and living with a partner), we fit a series of logistic regression models using the indicator as the outcome and the scaled versions of the HEART scales as predictors (Table 4). We found that after controlling for all the HEART scores, a one-standard deviation in the PAC score was associated with a 11% increase in the odds of experiencing any form of IPV. Also, a one-standard deviation in the PR score is associated with 14.5% higher odds of experiencing IPV and 10% lower odds of full disclosure to their partners.

**Table 4:** Unadjusted and adjusted logistic regression models of HEART scores on (1) reports of any IPV and (2) disclosure of ring and study to partner.

	Reported experiencing any IPV		Disclosure of ring and study to partner	
	Unadjusted OR (SE)	Adjusted OR (SE)	Unadjusted OR (SE)	Adjusted OR (SE)
Traditional Values (TV)	1.02(0.02)	1.00(0.03)	1.00(0.02)	1.02(0.02)
Partner Support (PS)	0.95* (0.03)	1.02 (0.06)	1.05** (0.02)	1.06* (0.04)
Partner Abuse & Control (PAC)	1.12*** (0.04)	1.11* (0.07)	0.98 (0.03)	1.07 (0.05)
Partner Resistance to HIV Prevention (PR)	1.15*** (0.05)	1.14** (0.07)	0.91** (0.04)	0.90* (0.05)
HIV Prevention Readiness (HPR)	1.01 (0.13)	1.40 (0.34)	1.10 (0.09)	1.02 (0.1)

* Associated probability value (p) < 0.1

** p < 0.05

***p < 0.01.

### Research question 3: How have HEART subscales been used to guide the CHARISMA pilot intervention?

During the CHARISMA pilot, counselors relied on both the HEART subscale scores, women’s reported disclosure to partners, and their own discretion to determine which intervention module(s) to offer. Most women (82%) received one module and 17% received two or more modules; over half of participants were offered and received the IPV module (Table 5).

**Table 5:** Number and % of women assigned to counseling modules, over follow-up.

Module	Baseline N = 95	1^st^ follow-upN = 91	2^nd^ follow-up N = 77
A: Partner communication	27 (28.4)	15 (16.5)	8 (10.4)
B: Disclosure	31 (32.6)	12 (13.2)	12 (15.6)
C: IPV	55 (57.9)	20 (22.0)	10 (32.3)

All but one woman who reported IPV at baseline were offered the IPV module based on their HEART scores. (The counselor overrode the IPV recommendation because this participant recently changed partners and was now in a more supportive relationship.) Additionally, almost half of women who did not report IPV were also offered this module. As would be expected, these women had significantly higher PAC and PR scores, and lower PS scores, than other women who did not report IPV and were assigned the communication or disclosure modules. Table 6 presents the means and differences in HEART scores by women who did and did not report any baseline IPV and who did and did not receive module C.

**Table 6:** Relationship between baseline HEART scores (PS, PAC and PR), baseline reports of IPV and receiving the IPV module.

	No IPV Reported at Baseline			IPV Reported at Baseline		
	No IPV module	IPV module assigned	Difference	No IPV module	IPV module assigned	Difference
*Baseline*	(n = 39)	(n = 44)		(n = 1)	(n = 11)	
PS	52.9	46.7	3.20***	54	43.0	11.0
PAC	10.4	17.9	‒6.22***	15	22.5	7.5
PR	6.4	9.2	‒2.96***	5	14.0	9.0
*Follow-up #1*	(n = 66)	(n = 13)		(n = 5)	(n = 7)	
PS	54.7	49.6	2.34**	48.4	40.6	1.2
PAC	11.1	14.1	‒2.68***	17.4	18.4	‒0.2
PR	6.0	6.3	‒0.43	8.4	11.4	‒0.7
*Follow-up #2*	(n = 58)	(n = 7)		(n = 9)	(n = 3)	
PS	55.4	48.4	2.82***	55.3	50.3	1.1
PAC	10.3	11.4	‒0.85	11.3	13.3	‒0.8
PR	5.9	9.1	‒2.46**	5.1	8.7	‒8.9***

* Associated probability value *p < 0.1

** p < 0.05

***p < 0.01. No statistical tests were conducted among women who reported IPV at baseline since only one woman in this group was not assigned the IPV module.

### Research question 4: How do HEART subscales perform over time?

**Fig 2:**
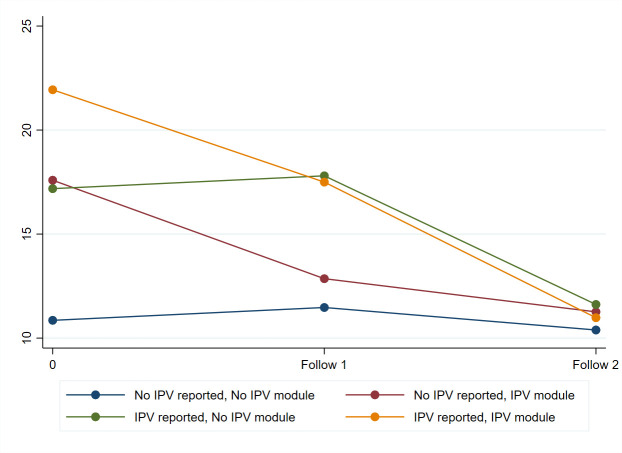
Model-predicted partner abuse & control score by baseline IPV status and whether they received the IPV counseling module.

Over time, the model-predicted scores for PAC (Fig 2), TV and PR (S1 and S3 Figs) decreased and the PS score (S2 Fig) increased among all participants. Women who reported any IPV at baseline had larger increases over time in PS, as well as decreases in PAC and PR, than women who did not report IPV. Receiving IPV counseling was associated with a decrease from baseline to follow-up 1 at month 3 in the PAC score, regardless of whether IPV was reported at baseline. Between follow-ups, the decrease was associated mostly with having reported IPV at baseline.

Fig 2 below shows the model-predicted change in PAC scores for groups of women who did and did not report IPV at baseline and were or were not assigned the IPV module. Note that women who received the IPV module showed steep declines in PAC scores, with women who reported IPV at baseline and received this module showing the steepest declines from baseline to the month 6 endline. As expected, the PAC scores were lowest at baseline and remained low over time among women who reported no IPV at baseline and did not receive the IPV module.

## Discussion

In this study, we evaluated the performance of a multi-dimensional tool called HEART to assess the quality of women’s primary relationship, including the level of partner support and the potential for conflict, to guide selection of counseling modules that might support PrEP use. Overall, the tool’s psychometric properties, including subscale intercorrelations and reliabilities, were similar in two different samples of South African women. In the pilot CHARISMA study, women reporting baseline IPV also had significantly higher scores on PAC and PR scales than women who did not report IPV at baseline. Women who had not fully disclosed to their partners about either/or both study participation and vaginal ring use had significantly lower PS and higher PR scores.

Although the HEART was not used exclusively to determine which counseling modules to offer women during the pilot CHARISMA intervention, those who received the IPV module reported significantly lower levels of PS and higher levels of PAC and PR than other women. This suggests that counselors were applying information from the HEART in a consistent way, even if recommending the IPV module more liberally than originally intended. Furthermore, the intermittent administration of HEART over the 6-month intervention facilitated prospective monitoring. Indeed, changes in HEART scores over time indicated improvements in relationship dynamics, with steep reductions in PAC scores for most women except those who had low baseline PAC scores, and the steepest declines among women who received the IPV module.

Despite some promising results, our study has several limitations. First, evidence of HEART validity has only been demonstrated among adult women recruited through a clinical trial setting in Johannesburg, SA. In the pilot intervention, almost all women had a primary sexual partner but less than 40% lived with their partner. It remains unclear how well the HEART tool will perform overall, or by individual subscale, among women in other geographic settings or among those who have different relationship contexts. Furthermore, while all but the HPR scale showed moderate to good psychometric properties across the two studies, only three of the five subscales provided partnership-related information that directly informed the intervention. In fact, the HPR scale continued to be strongly skewed, as identified in our first analysis [22]. Based on these results, we considered eliminating the HPR and TV scales from the tool prior to implementing the effectiveness study. However, interim evaluation of the pilot [31] suggested that the progress of constructs and items in the HEART evoked deeper, yet non-threatening reflection among participants of their relationship with a primary partner. This led us to revise and further study HEART constructs in the upcoming effectiveness study, rather than eliminate immediately. And, finally, the goal of the pilot CHARISMA intervention, of which HEART is a component, is to assist women to safely and effectively adhere to PrEP products. As yet, we are not able to evaluate whether HEART scores can prospectively identify women who face challenges adhering to PrEP use.

In a final expansion phase of the CHARIMSA project, a randomized controlled trial aims to evaluate the effectiveness of the CHARISMA intervention in the context of oral PrEP provision. As part of this expansion study, we will address several remaining questions related to HEART. One question is whether it is possible to improve the HPR subscale [22] by revising subscale content or whether the subscale should be removed from HEART. From a feasibility perspective, a smaller tool with fewer items would take less time to implement in a busy clinic setting. In an interim assessment of CHARISMA acceptability and feasibility, most participants (61%) felt the tool took the right amount of time, while 25% felt it was too long [31]. On the other hand, some lay counselors noted that the flow of the tool, which moves from more normative items related to gender-based values to partner support, partner abuse and control, resistance towards HIV prevention and finally HIV prevention readiness facilitated participants’ ability to think carefully about their current relationship. A shorter and more focused tool might be experienced as too abrupt and not reflective of participants’ nuanced relationships. Another linger question is whether to narrow the range of scores that would lead to a decision to offer the IPV module. The goal is to ensure that the tool is both sensitive to identifying women who could face partner-related challenges to adherence, but also targeted to those who will benefit from IPV or disclosure modules without distressing clients or overburdening counselors. During the interim assessment, some women felt that they had been incorrectly identified as being at high-risk for IPV and objected to receiving the IPV module [31].

There are several ethical issues with using a tool to guide clinical decisions related to IPV counseling. First, it is likely preferable to offer IPV counseling and resources to women who do not need it rather than miss offering them to women who might not be comfortable reporting it. However, such counseling can be very resource and time-intensive, particularly if the content does not address a recipient’s life context. A second issue relates to measurement. Given the stigma of directly disclosing IPV [32, 33], we designed a tool that used a less direct route to identify potential IPV. Indeed, the multi-dimensional HEART tool successively orients women to think through their relationship by moving through a series of statements related to traditional values and partner support before moving into items related to partner control and abuse. Additionally, the ability to indicate a level of agreement rather than a binary “yes or no” outcome may also make it easier to form women to acknowledge relationship concerns. Second, in the pilot we applied a fairly broad range of PAC scores to module C offering. Informal feedback from pilot intervention counselors indicated that some women whose scores suggested they receive the IPV module strongly disagreed with that assessment, leading us to conclude that over-prescribing the IPV intervention may also have its ethical challenges. An important take-home from the pilot study was the importance of using such decision-tools flexibly with final decisions about which module to offer remaining with the counselor or clinician.

In the Effectiveness Study, we will determine the degree to which the HEART and/or specific subscales help intervene to support partner communication, disclosure and negotiation of PrEP and ultimately lead to better adherence.

## Conclusions

These data offer preliminary evidence for HEART construct and predictive validity and support its further evaluation to guide implementation and monitor the impact of the CHARISMA intervention in the randomized controlled study.

## Supporting information

S1 Table Various measures of reliability for survey and pilot samples. Raw Alpha: Cronbach Alpha (based upon the covariances); Std Alpha: Standardized Alpha (based upon the correlations); G6: Guttman’s Lambda 6 reliability.
(DOCX)

S1 Fig Model-predicted traditional values score by baseline IPV status and whether they received the IPV counseling module. (TIF)

S2 Fig Model-predicted partner support score by baseline IPV status and whether they received the IPV counseling module. (TIF)

S3 Fig Model-predicted partner resistance score by baseline IPV status and whether they received the IPV counseling module. (TIF)

## Abbreviations

AGYW: Adolescent girls and young women
CHARISMA: Community Health clinic model for Agency in Relationships and Safer Microbicide Adherence
FTP: Former trial participant
HEART: Healthy Relationship Assessment Tool
HPR: HIV prevention readiness subscale
IPV: Intimate partner violence
MTN: Microbicides Trial Network
PAC: Partner abuse and control subscale
PR: Partner resistance to HIV prevention subscale
PrEP: Pre-exposure prevention prophylaxis
PS: Partner support subscale
TNP: trial-naïve participant
TV: Traditional values subscale
