# Supplementary material for: Evidence for use of a healthy relationships assessment tool in the CHARISMA pilot study
Source: PLoS One. 2021 Dec 31;16(12):e0261526. doi: 10.1371/journal.pone.0261526 (PMC8719706; doi:10.1371/journal.pone.0261526)
Supplement: S1 Table — Raw Alpha: Cronbach Alpha (based upon the covariances); Std Alpha: Standardized Alpha (based upon the correlations); G6: Guttman’s Lambda 6 reliability. (DOCX) [file pone.0261526.s001.docx]

|  | Survey | | | Pilot | | |  |
| --- | --- | --- | --- | --- | --- | --- | --- |
|  | Raw Alpha | Std Alpha | G6 | Raw Alpha | Std Alpha | G6 | |
| Traditional Values (TV) | 0.84 | 0.85 | 0.85 | 0.85 | 0.85 | 0.89 | |
| Partner Support (PS) | 0.81 | 0.81 | 0.80 | 0.80 | 0.81 | 0.82 | |
| Partner Abuse & Control (PAC) | 0.81 | 0.82 | 0.83 | 0.74 | 0.76 | 0.80 | |
| Partner Resistance to HIV Prevention (PR) | 0.80 | 0.80 | 0.78 | 0.83 | 0.85 | 0.87 | |
| HIV Prevention Readiness (HPR) | 0.68 | 0.72 | 0.68 | 0.56 | 0.53 | 0.54 | |
|  | | | | | | |  |
