# Supplementary figures and images for: Evidence for use of a healthy relationships assessment tool in the CHARISMA pilot study
Source: PLoS One. 2021 Dec 31;16(12):e0261526. doi: 10.1371/journal.pone.0261526 (PMC8719706; doi:10.1371/journal.pone.0261526)

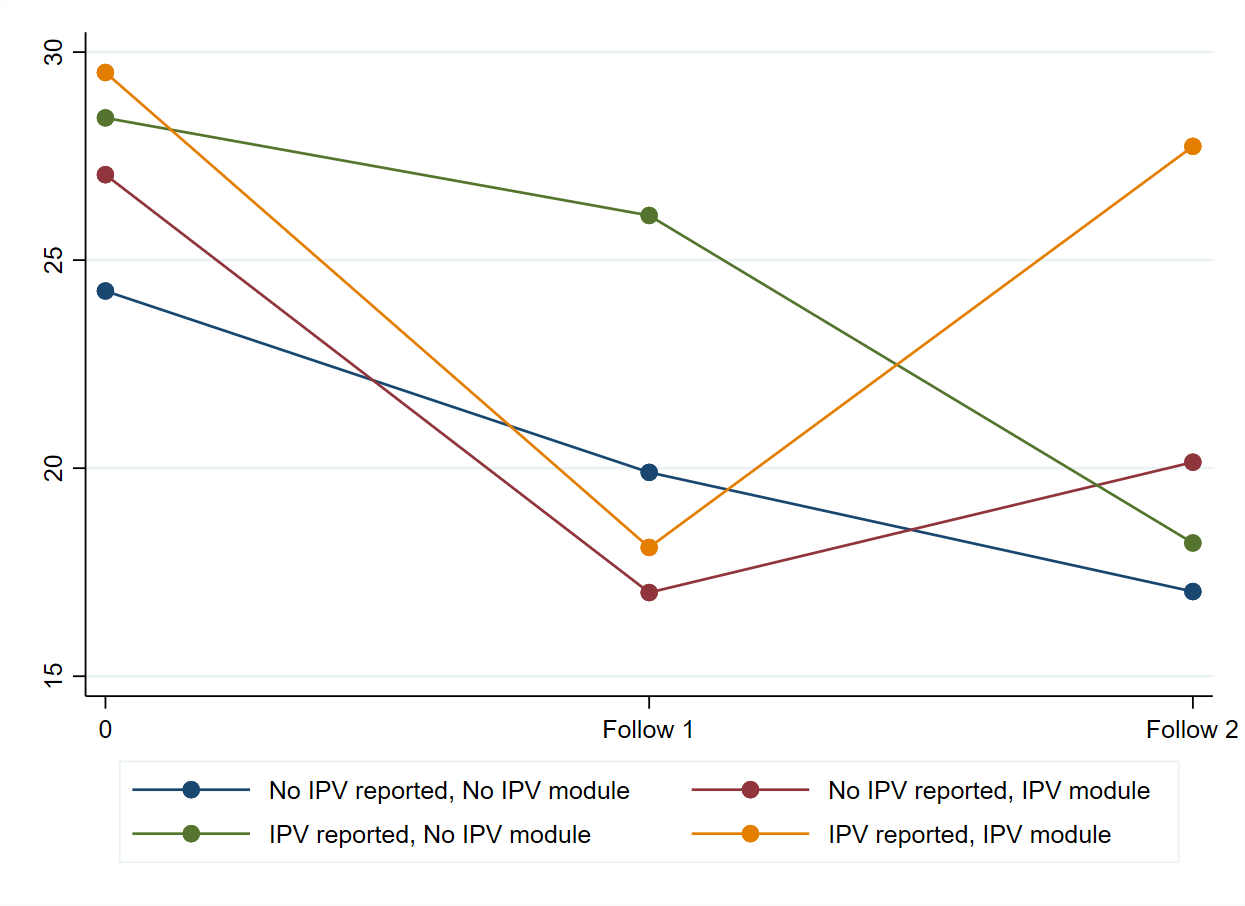

Supplement: S1 Fig — (TIF) [file pone.0261526.s002.tif]

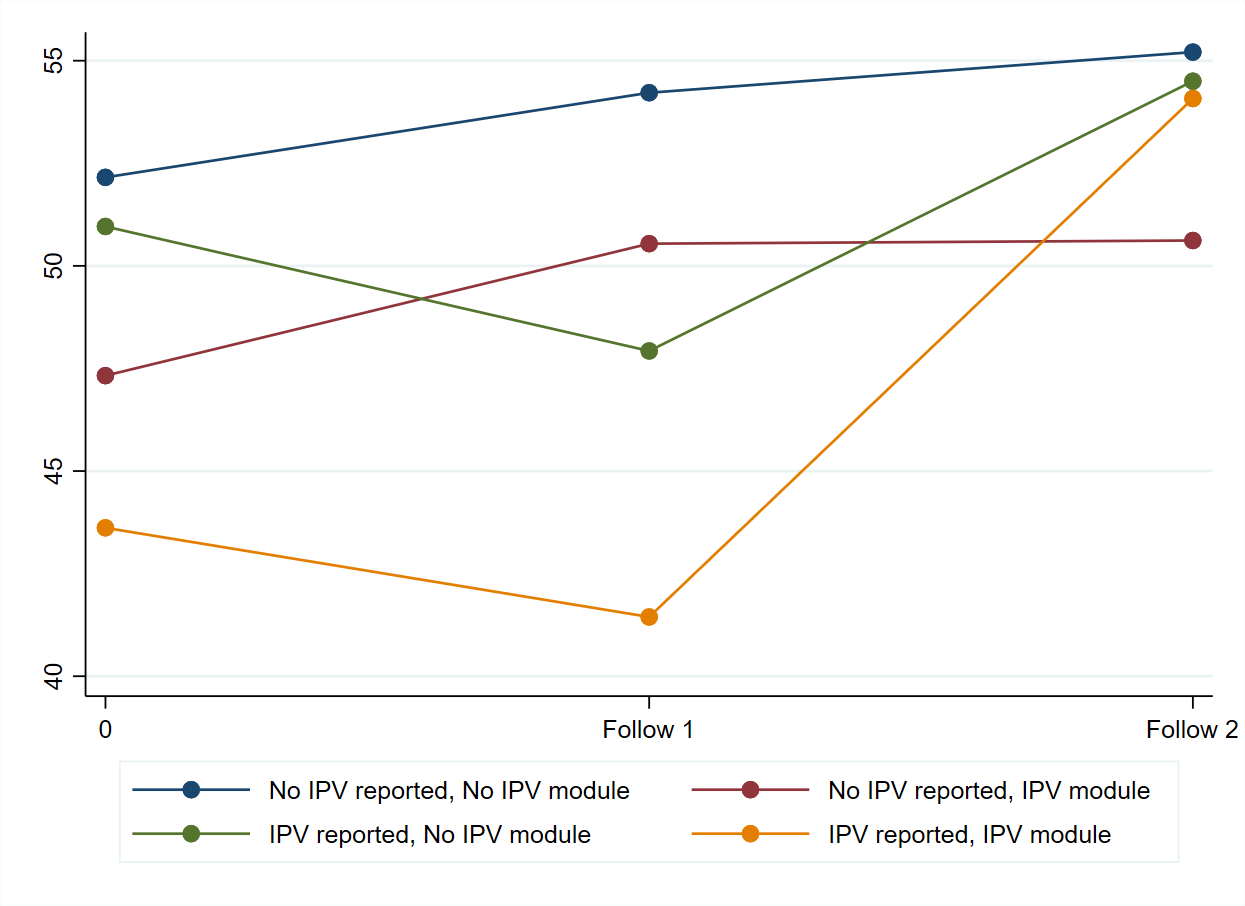

Supplement: S2 Fig — (TIF) [file pone.0261526.s003.tif]

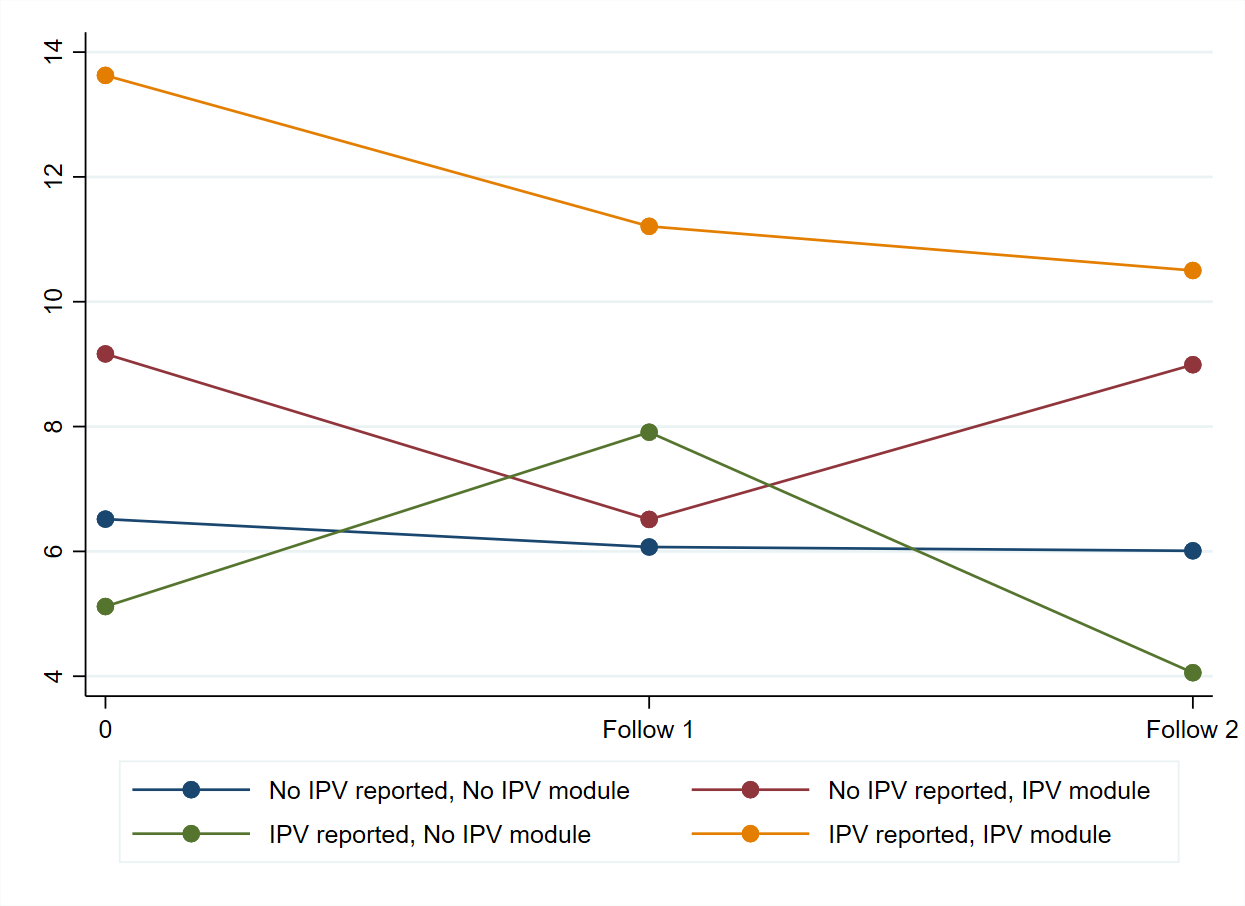

Supplement: S3 Fig — (TIF) [file pone.0261526.s004.tif]
